# Draft Genome Sequence of *Clostridium tyrobutyricum* Strain UC7086, Isolated from Grana Padano Cheese with Late-Blowing Defect

**DOI:** 10.1128/genomeA.00614-13

**Published:** 2013-08-15

**Authors:** Daniela Bassi, Cecilia Fontana, Simona Gazzola, Ester Pietta, Edoardo Puglisi, Fabrizio Cappa, Pier Sandro Cocconcelli

**Affiliations:** Istituto di Microbiologia, Università Cattolica del Sacro Cuore, Piacenza-Cremona, Italy

## Abstract

*Clostridium tyrobutyricum* is considered the main agent of late-blowing defect in the production of hard cheese. Here, we described the draft genome sequences and annotation of *C. tyrobutyricum* strain UC7086, which was isolated from Grana Padano cheese with blowing defect, and *C. tyrobutyricum* DSM 2637 type strain in a comparative study.

## GENOME ANNOUNCEMENT

Butyric clostridia are spore-forming anaerobic bacteria affecting the dairy industry by causing late-blowing defect, a particular kind of food spoilage in hard and semihard cheeses, such as Grana Padano, Parmigiano Reggiano, Emmental, and Gouda (1–6). Among them, *Clostridium tyrobutyricum* is considered the main organism that is responsible for this problem (3, 4). Its spores, which contaminate milk and are resistant to whole-cheese manufacturing, germinate during ripening, and the butyric fermentation of the vegetative cells causes the production of butyric acid, acetic acid, hydrogen, and CO_2_, bursting of cheese paste, and a consequent undesirable taste. Recently, *C. tyrobutyricum* obtained great attention for biofuel, acetic acid, and butanol production (7, 8). A deeper investigation of its metabolic pathways and adaptation mechanisms can help to understand its negative and positive effects in food production and industrial applications.

In this work, a *de novo* shotgun sequencing of *C. tyrobutyricum* strain UC7086, isolated from Grana Padano cheese with blowing defect, and of *C. tyrobutyricum* DSM 2637 type strain has been performed. The genomes were sequenced using an Illumina HiSeq 1000 platform from the Functional Genomics Centre, Scientific and Technological Department of the University of Verona. Quality-filtered reads were assembled using the Velvet software (version 1.1.04) (9), and contig sequences were annotated in the RAST server (10). A 3,064,215-bp assembly was obtained for *C. tyrobutyricum* UC7086, consisting of a total of 110 contigs and with a mean G+C content of 31%. The type strain *C. tyrobutyricum* DSM 2637 has 3,007,342 bases and was assembled in 175 contigs with a mean G+C content of 30.6%. The annotated contigs contain 3,038 putative coding sequences (CDSs) and 51 predicted RNAs for *C. tyrobutyricum* UC7086 and 3,066 CDSs and 41 predicted RNAs for *C. tyrobutyricum* DSM 2637. Loaded in the RAST server, the reported genomes of UC7086 and DSM 2637 contain 350 and 365 subsystems, respectively, which constitute the basis for creating the *C. tyrobutyricum* metabolic network.

A comparative genome analysis between both *C. tyrobutyricum* deep-sequenced genomes revealed an overall high protein sequence identity. A total of 29 protein-coding genes were unique in *C. tyrobutyricum* UC7086 and 35 in DSM 2637. Strain UC7086 has genes for proteins that are involved in amino acid metabolism, which reveal a possible adaptation to the cheese environment during ripening (lysine and proline uptake and degradation and arginine and ornithine degradation), DNA metabolism (CRISPR-associated proteins and restriction-modification systems), and carbohydrate metabolism (mannose and mannitol utilization). Particularly, the presence in UC7086 of a gene cluster for urea decomposition (*ureA*, *ureC*, *ureD*, *ureE*, *ureF*, and *ureG*) with consequent CO_2_ production might be considered an adaption strategy and a stress response mechanism in hard cheese. In the DSM 2637 strain, 35 singular genes were related to carbohydrate metabolism (maltose and xylose utilization), phage packaging machinery, replication and introns (helicase, terminase), spore germination (*gerKB*), and motility (*fliH*).

Further analyses are in progress to better understand the annotated genome sequence data, as well as the gap closure to complete the present draft genome. This information will be useful to compare the genomes of different *C. tyrobutyricum* strains to one another and to those of other *Clostridium* species.

### Nucleotide sequence accession numbers.

This whole-genome shotgun project has been deposited at DDBJ/EMBL/GenBank under the accession no. ANOE00000000 for *C. tyrobutyricum* UC7086 and ARYO00000000 for *C. tyrobutyricum* DSM 2637. The versions described in this paper are the first versions, with accession no. ANOE01000000 and ARYO01000000.
